# Supplementary material for: Clonal hematopoiesis of indeterminate potential, DNA methylation, and risk for coronary artery disease
Source: Nat Commun. 2022 Sep 12;13:5350. doi: 10.1038/s41467-022-33093-3 (PMC9468335; doi:10.1038/s41467-022-33093-3)
Supplement: Supplementary file 1 — Supplementary Information [file 41467_2022_33093_MOESM1_ESM.pdf]

Supplementary Note 1

DNA methylation data

DNAm data preparation and quality control in CHS was previously described in Agha, et al. [1]. Briefly, the minfi R package[2] was used for DNAm quality control. Samples were excluded if (i) median intensities across the methylated and unmethylated channels were <10.5 (log2), (ii) >0.5% of probes failed detection, (iii) QC probes fell >3 SD from the mean, (iv) sample swaps appeared likely due to sex mismatches or genotype inconsistency with prior genotyping. Subset-quantile Within Array Normalization (SWAN)[3] was used to standardize the DNAm values, and the Houseman, et al. [4] method was used to estimate blood cell proportions from the DNAm data.

Detailed information on the DNAm assay and quality control procedures used in ARIC was reported previously[5]. In brief, individuals were excluded from the analyses if the pass rate for the DNA sample for the participant was <95% (number of probes with a detection p-value <0.01/number of probes on the array), if sex mismatch appeared likely based on principal component analysis, or if genotypes for 24 single nucleotide polymorphisms present on the array were inconsistent with prior genotyping. CpG sites were excluded if >5% of the samples showed a detection p-value >0.01, or if the average detection p-value for a Y chromosome CpG was >0.01 among males. Methylation values were normalized using Noob background and dye bias correction[6] and the Beta Mixture Quantile dilation (BMIQ) method[7].

Expanded CHIP Analysis

We also performed EWAS for expanded CHIP (VAF>10%) in CHS, and identified 7881 CpGs at FDR <0.05, 560 CpGs were Bonferroni-significant ($P<1.04\times10^{-7}$) (Supplementary Fig. 5a, b). At FDR<0.05, presence of expanded CHIP was associated with decreasing DNAm at 0.95% (4546 / 478661) of sites and increasing DNAm at 0.70% (3335) of sites. The genomic inflation factor was 1.40 in expanded CHIP meta EWAS (Supplementary Fig. 5c). Of the 7881 CpGs, ~54% (4226) were replicated with FDR<0.05 and concordant effect direction in the multi-ancestry meta-analysis of ARIC-AA and ARIC-EA EWAS. Summary statistics for all 4226 replicated expanded CHIP

associated CpGs from the discovery, replication, and combined meta-analysis of EWAS is presented in Supplementary Data 9.

Enrichment Analysis

We performed one-sided Fisher's exact test to identify genes with ≥10 replicated CpGs with OR>1 and $P<0.05$. We observed ten or more replicated CpGs near few genes which was more than expected simply by chance ($P<0.05$; see Supplementary Table 4). For example, presence of any CHIP, expanded CHIP or *DNMT3A* CHIP was associated with reduced DNAm in ≥21 CpGs annotated to *PRDM16* (OR≥3.8; $P\leq4.7\times10^{-7}$), ≥13 CpGs in *PPT2-PRRT1* (OR≥14.6; $P\leq1.6\times10^{-11}$) and ≥12 CpGs in *VARS* (OR≥14.7; $P\leq1.8\times10^{-10}$). Similarly, presence of any CHIP and expanded CHIP was associated with reduced DNAm in ≥13 CpGs in *AGAP2* (OR≥50.2; $P\leq5.9\times10^{-17}$). Presence of *DNMT3A* CHIP was associated with reduced DNAm in 12 CpGs in *HOXB3* (OR=35; $P=2.0\times10^{-14}$). Finally, presence of *TET2* CHIP was associated with increased DNAm in 18 CpGs in *RPTOR* (OR=14.1; $P=6.1\times10^{-15}$), 15 CpGs in *HDAC4* (OR=12.1; $P=8.4\times10^{-12}$), and 10 CpGs in *TRIM39* (OR=28.2; $P=9.9\times10^{-12}$).

Supplementary Figures

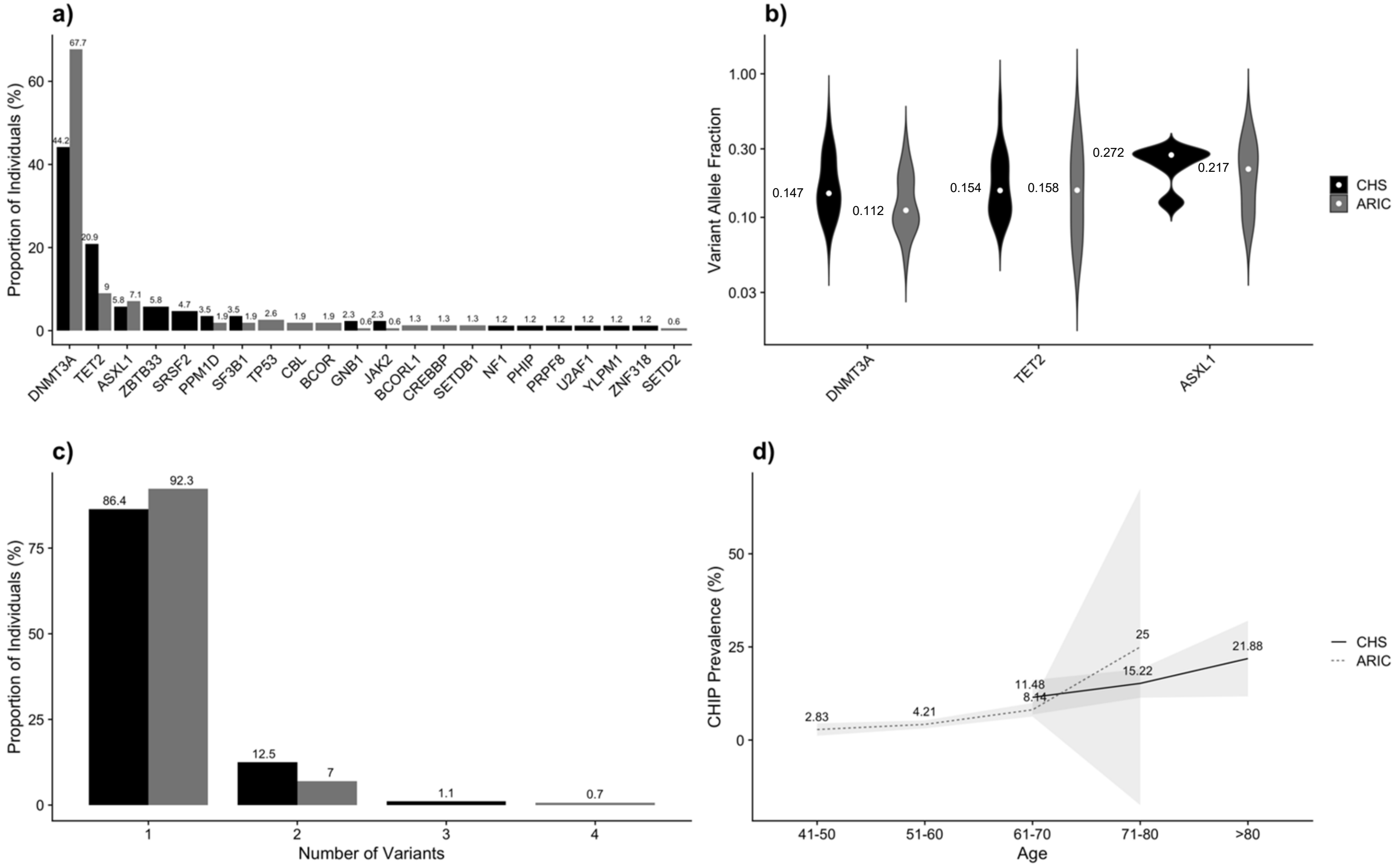

Supplementary Figure 1: Distribution of CHIP in CHS and ARIC study participants. (a) CHIP counts by gene detected in the two studies. (b) Distribution of variant allele fraction in top CHIP driver genes, with median VAF represented by white circles and printed to the left of each violin plot. (c) Distribution of number of CHIP variants per individual, with proportions represented by bar height. (d) CHIP prevalence by cohort.

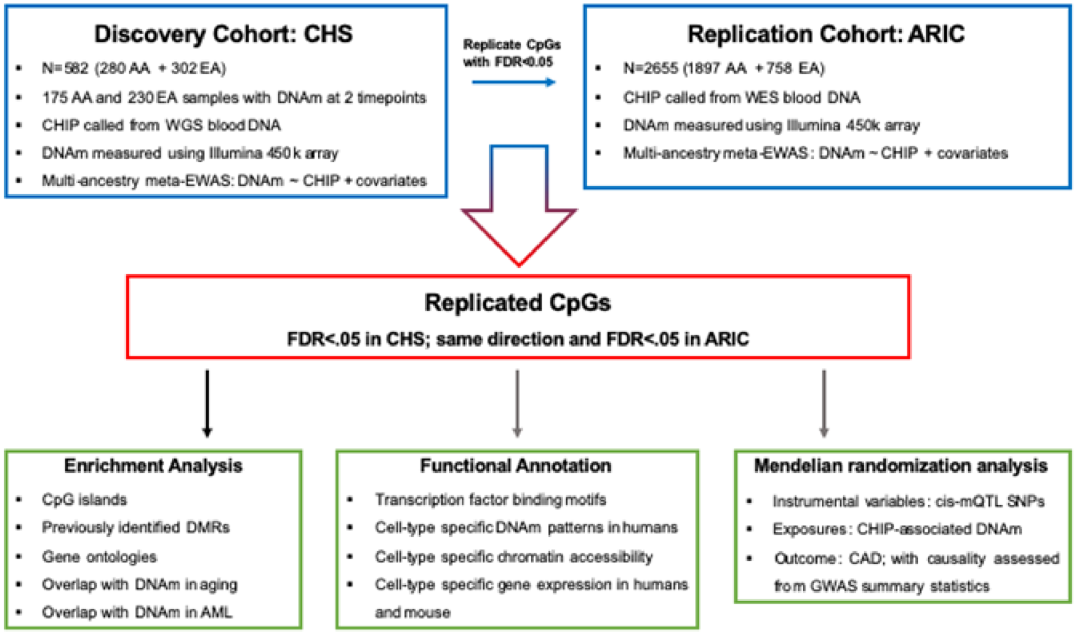

Supplementary Figure 2: Workflow of the CHIP EWAS and meta-analysis. Each ancestry-stratified EWAS was adjusted for age, age^2, sex, batch, and estimated cell type proportions. The CHS EWAS was also adjusted for longitudinal DNAm measures using random individual effects. Inverse-variance-weighted fixed effect meta-analysis was performed using METAL software[8]. CHS: Cardiovascular Health Study; ARIC: Atherosclerosis Risk in Communities; AA: African ancestry; EA: European ancestry. DMR: differentially methylated region.

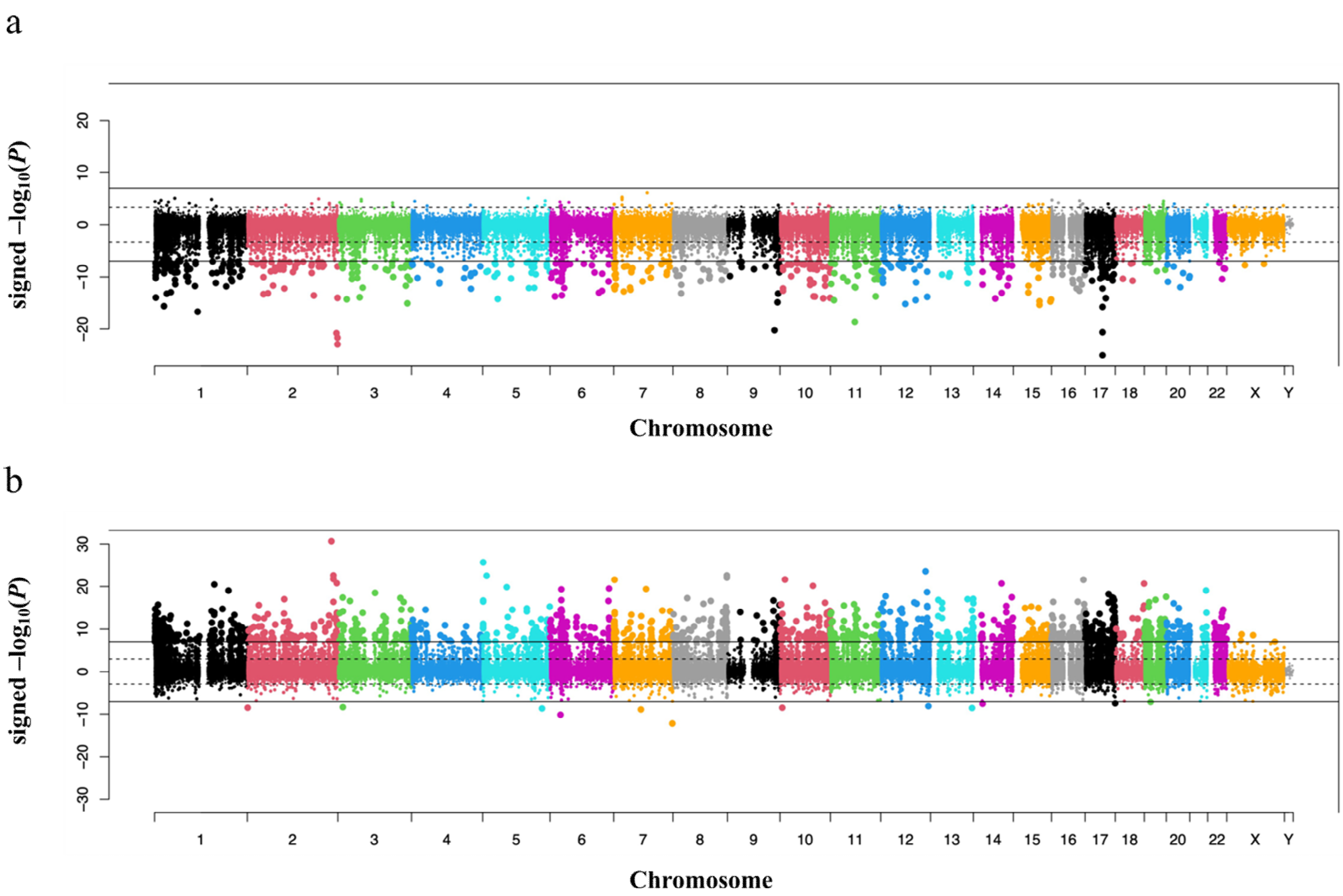

Supplementary Figure 3: Manhattan plot of discovery multi-ancestry meta-EWAS for (a) *DNMT3A* and (b) *TET2* CHIP and DNA methylation in CHS cohort. Directional Manhattan plot, where direction indicates positive vs. negative correlations between CHIP and DNAm. Each dot represents a CpG site, with genomic location on the x-axis and $-\log_{10}(P)$*sign(test statistic) on the y-axis, where P values are based on two-sided inverse-variance-weighted meta-analysis. Solid horizontal line indicates Bonferroni significance, and dashed line indicates 5% FDR. (a) *DNMT3A* CHIP: 499 CpGs at Bonferroni and 4,528 CpGs at FDR<0.05 threshold; (b) *TET2* CHIP: 1,595 CpGs at Bonferroni and 11,805 CpGs at FDR<0.05 threshold.

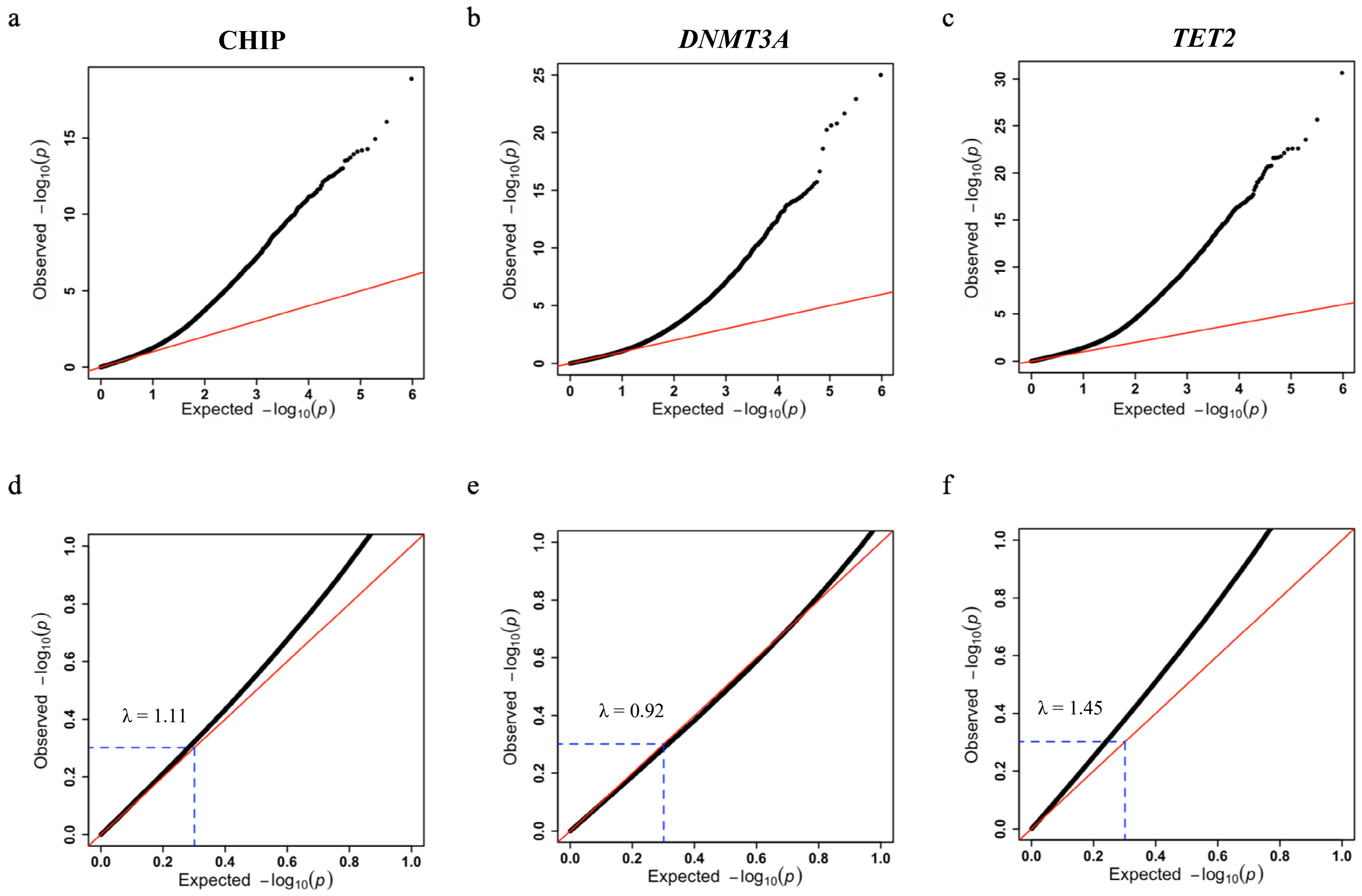

Supplementary Figure 4: Quantile-quantile plots of expected and observed *-log₁₀(P)* in CHIP meta EWAS in CHS. Upper plots show full range while lower plots are zoomed in to show range surrounding the expected median of the distribution (median($-\log_{10}(P)$) = $-\log_{10}(0.5) = 0.30103$; blue dotted line). Genomic inflation factors computed as ratio of observed to expected median χ^2 quantiles after conversion of p-values to $\chi^2(1)$ quantiles. (a,d) any CHIP: $\lambda = 1.11$, (b,e) *DNMT3A* CHIP: $\lambda = 0.92$, and (c,f) *TET2* CHIP: $\lambda = 1.45$.

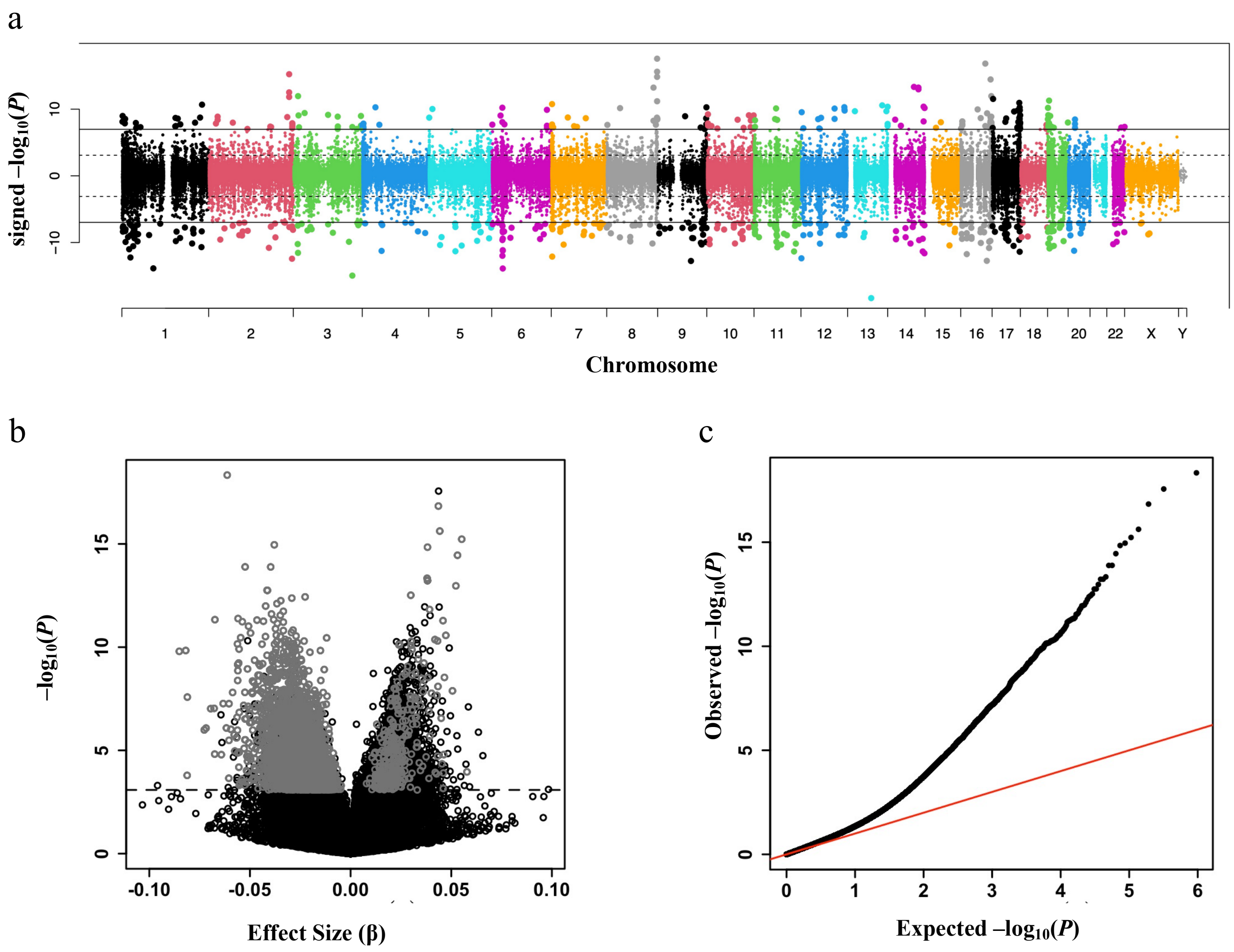

Supplementary Figure 5: Discovery EWAS for expanded CHIP (variant allele fraction>10%) in CHS. (a) Directional Manhattan plot, where direction indicates positive vs. negative correlations between expanded CHIP and DNAm. Each dot represents a CpG site, with genomic location on the x-axis and $-\log_{10}(P)$*sign(test statistic) on the y-axis, where *P* values are based on two-sided inverse-variance-weighted meta-analysis. Solid horizontal line indicates Bonferroni significance, and dashed line indicates 5% FDR. 560 CpGs pass Bonferroni and 7,881 CpGs pass FDR<0.05 threshold. (b) Volcano plot depicting the effect size and *-log*$_{10}$*(P)* from CHS meta EWAS of expanded CHIP. Dashed line indicates FDR <0.05, and the colors highlight replicated CpGs. (c) Quantile-quantile plot of expected and observed *-log*$_{10}$*(P)*: $\lambda = 1.40$.

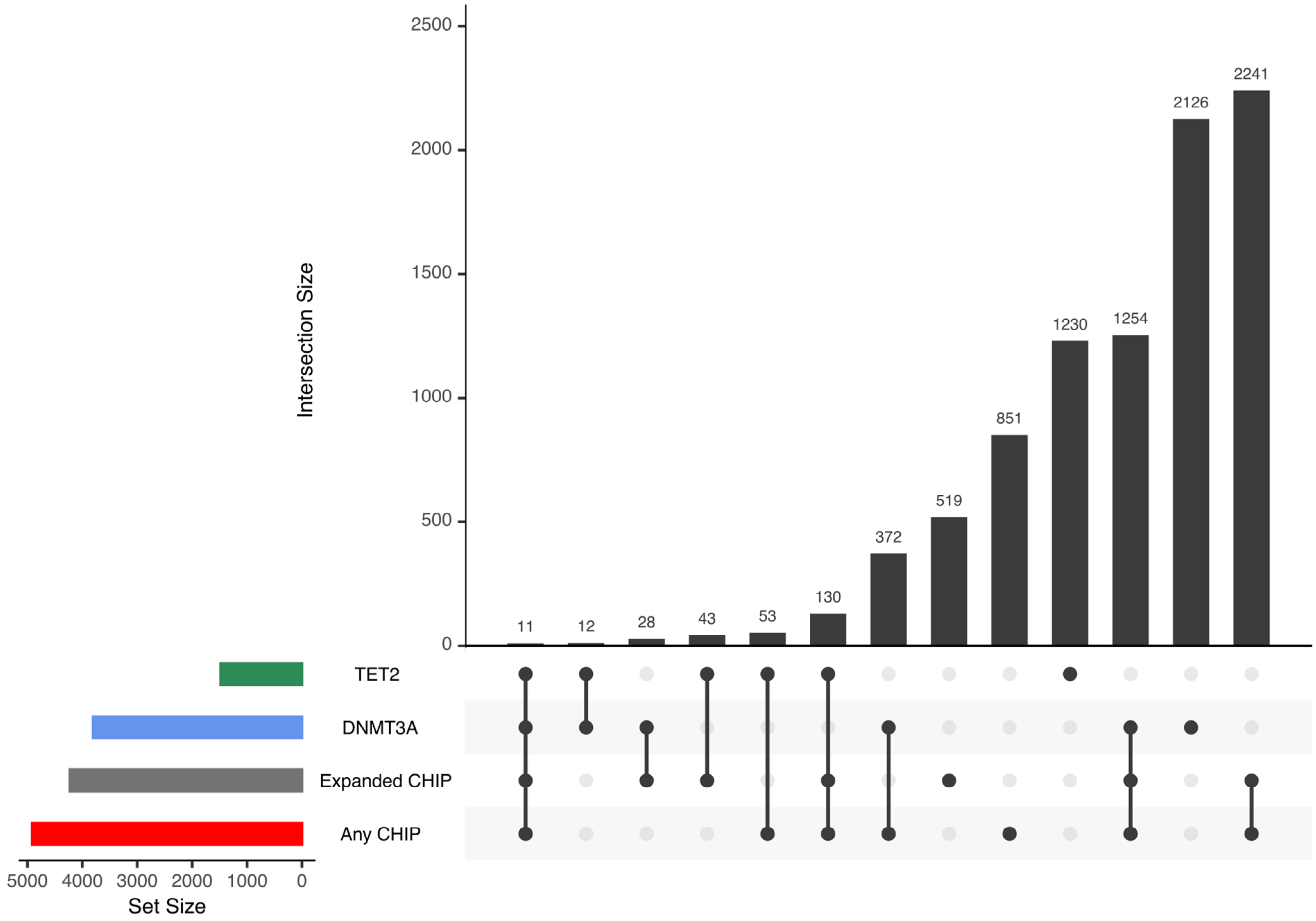

Supplementary Figure 6: UpSet[9] plot showing the intersection between sets of CpGs associated with four categories of CHIP X-axis: intersection between sets of replicated CpGs associated with four categories of CHIP mutations; y-axis: count of CpGs.

Homer Known Motif Enrichment Results for 200-bp regions surrounding *DNMT3A*-associated CpG sites

Total Target Sequences = 3526, Total Background Sequences = 402448 (after removal of overlapping sequences)

Rank	Motif	Name	P-value	log P-pvalue	q-value (Benjamini)	# Target Sequences with Motif	% of Targets Sequences with Motif	# Background Sequences with Motif	% of Background Sequences with Motif
1	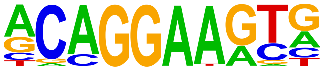	ERG(ETS)/VCaP-ERG-ChIP-Seq(GSE14097)/Homer	1e-23	-5.504e+01	0.0000	834.0	23.65%	68053.9	16.91%
2	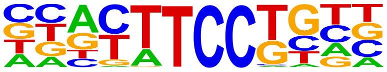	Etv2(ETS)/ES-ER71-ChIP-Seq(GSE59402)/Homer(0.967)	1e-20	-4.673e+01	0.0000	499.0	14.15%	37287.9	9.27%
3	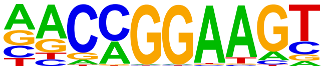	ETV1(ETS)/GIST48-ETV1-ChIP-Seq(GSE22441)/Homer	1e-15	-3.621e+01	0.0000	692.0	19.62%	58601.4	14.56%
4	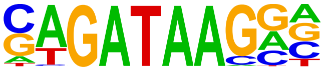	Gata1(Zf)/K562-GATA1-ChIP-Seq(GSE18829)/Homer	1e-13	-3.053e+01	0.0000	235.0	6.66%	16021.2	3.98%
5	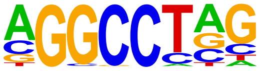	ZNF711(Zf)/SHSY5Y-ZNF711-ChIP-Seq(GSE20673)/Homer	1e-12	-2.956e+01	0.0000	1056.0	29.94%	98684.2	24.52%
6	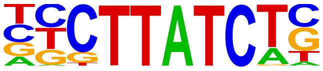	Gata2(Zf)/K562-GATA2-ChIP-Seq(GSE18829)/Homer	1e-12	-2.944e+01	0.0000	253.0	7.17%	17809.4	4.43%
7	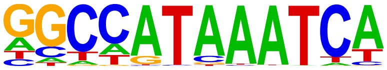	Hoxc9(Homeobox)/Ainv15-Hoxc9-ChIP-Seq(GSE21812)/Homer	1e-12	-2.865e+01	0.0000	195.0	5.53%	12812.1	3.18%
8	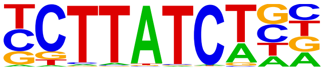	Gata6(Zf)/HUG1N-GATA6-ChIP-Seq(GSE51936)/Homer	1e-12	-2.832e+01	0.0000	327.0	9.27%	24838.7	6.17%
9	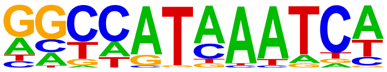	HOXA9(Homeobox)/HSC-Hoxa9-ChIP-Seq(GSE33509)/Homer	1e-11	-2.743e+01	0.0000	243.0	6.89%	17260.0	4.29%
10	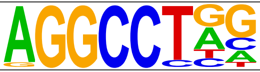	ZFX(Zf)/mES-Zfx-ChIP-Seq(GSE11431)/Homer	1e-11	-2.691e+01	0.0000	794.0	22.51%	71990.9	17.89%
11	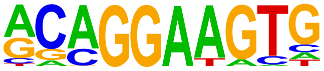	ETS1(ETS)/Jurkat-ETS1-ChIP-Seq(GSE17954)/Homer	1e-11	-2.664e+01	0.0000	505.0	14.32%	42534.6	10.57%
12	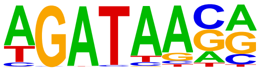	GATA3(Zf)/iTreg-Gata3-ChIP-Seq(GSE20898)/Homer	1e-10	-2.434e+01	0.0000	486.0	13.78%	41299.8	10.26%
13	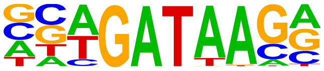	Gata4(Zf)/Heart-Gata4-ChIP-Seq(GSE35151)/Homer	1e-10	-2.371e+01	0.0000	350.0	9.92%	28080.0	6.98%
14	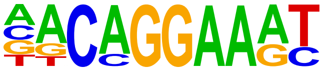	EWS:FLI1-fusion(ETS)/SK_N_MC-EWS:FLI1-ChIP-Seq(SRA014231)/Homer	1e-9	-2.281e+01	0.0000	298.0	8.45%	23303.9	5.79%
15	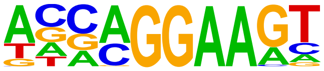	EHF(ETS)/LoVo-EHF-ChIP-Seq(GSE49402)/Homer	1e-9	-2.240e+01	0.0000	566.0	16.05%	50001.4	12.42%
16	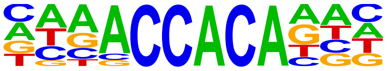	RUNX2(Runt)/PCa-RUNX2-ChIP-Seq(GSE33889)/Homer	1e-8	-2.070e+01	0.0000	328.0	9.30%	26707.8	6.64%
17	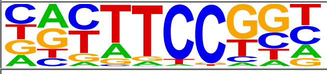	Fli1(ETS)/CD8-FLI-ChIP-Seq(GSE20898)/Homer	1e-8	-1.910e+01	0.0000	558.0	15.82%	50336.1	12.51%
18	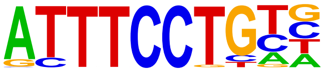	EWS:ERG-fusion(ETS)/CADO_ES1-EWS:ERG-ChIP-Seq(SRA014231)/Homer	1e-8	-1.863e+01	0.0000	315.0	8.93%	25990.0	6.46%
19	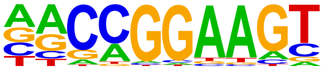	GABPA(ETS)/Jurkat-GABPa-ChIP-Seq(GSE17954)/Homer	1e-7	-1.786e+01	0.0000	430.0	12.19%	37685.8	9.36%
20	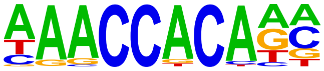	RUNX1(Runt)/Jurkat-RUNX1-ChIP-Seq(GSE29180)/Homer	1e-7	-1.733e+01	0.0000	403.0	11.43%	35132.2	8.73%

21	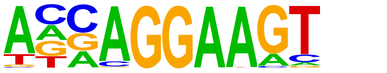	ELF3(ETS)/PDAC-ELF3-ChIP-Seq(GSE64557)/Homer	1e-7	-1.679e+01	0.0000	311.0	8.82%	26111.2	6.49%
22	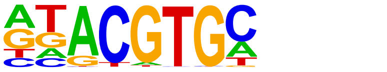	HIF-1b(HLH)/T47D-HIF1b-ChIP-Seq(GSE59937)/Homer	1e-6	-1.591e+01	0.0000	509.0	14.43%	46484.6	11.55%
23	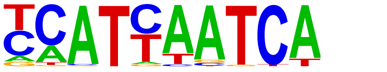	Pdx1(Homeobox)/Islet-Pdx1-ChIP-Seq(SRA008281)/Homer	1e-6	-1.587e+01	0.0000	293.0	8.31%	24599.0	6.11%
24	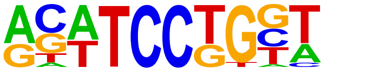	SPDEF(ETS)/VCaP-SPDEF-ChIP-Seq(SRA014231)/Homer	1e-6	-1.548e+01	0.0000	450.0	12.76%	40563.5	10.08%
25	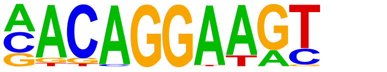	Ets1-distal(ETS)/CD4+-PolII-ChIP-Seq(Barski_et_al.)/Homer	1e-5	-1.281e+01	0.0000	138.0	3.91%	10452.7	2.60%
26	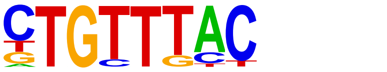	Foxo1(Forkhead)/RAW-Foxo1-ChIP-Seq(Fan_et_al.)/Homer	1e-5	-1.272e+01	0.0000	694.0	19.68%	67436.6	16.76%
27	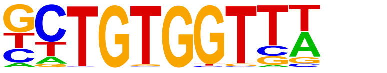	RUNX-AML(Runt)/CD4+-PolII-ChIP-Seq(Barski_et_al.)/Homer	1e-5	-1.222e+01	0.0001	280.0	7.94%	24441.5	6.07%
28	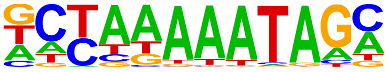	Mef2c(MADS)/GM12878-Mef2c-ChIP-Seq(GSE32465)/Homer	1e-5	-1.204e+01	0.0001	138.0	3.91%	10617.0	2.64%
29	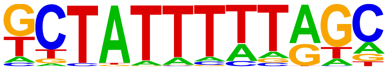	Mef2d(MADS)/Retina-Mef2d-ChIP-Seq(GSE61391)/Homer	1e-5	-1.198e+01	0.0001	71.0	2.01%	4586.9	1.14%
30	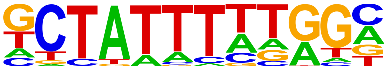	Mef2b(MADS)/HEK293-Mef2b.V5-ChIP-Seq(GSE67450)/Homer	1e-4	-1.151e+01	0.0001	256.0	7.26%	22268.9	5.53%
31	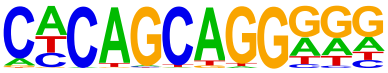	Unknown-ESC-element(?)/mES-Nanog-ChIP-Seq(GSE11724)/Homer	1e-4	-1.149e+01	0.0001	334.0	9.47%	30182.6	7.50%
32	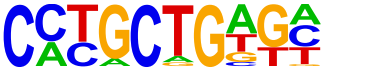	Zic(Zf)/Cerebellum-ZIC1.2-ChIP-Seq(GSE60731)/Homer	1e-4	-1.135e+01	0.0001	410.0	11.62%	38089.0	9.46%
33	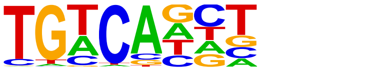	Tgif2(Homeobox)/mES-Tgif2-ChIP-Seq(GSE55404)/Homer	1e-4	-1.133e+01	0.0001	1138.0	32.27%	116681.2	28.99%
34	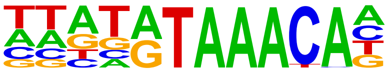	Foxf1(Forkhead)/Lung-Foxf1-ChIP-Seq(GSE77951)/Homer	1e-4	-1.124e+01	0.0001	274.0	7.77%	24170.2	6.01%
35	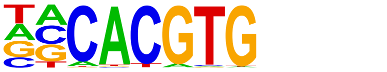	NPAS(bHLH)/Liver-NPAS-ChIP-Seq(GSE39860)/Homer	1e-4	-1.062e+01	0.0003	592.0	16.78%	57646.3	14.32%
36	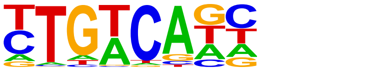	Tgif1(Homeobox)/mES-Tgif1-ChIP-Seq(GSE55404)/Homer	1e-4	-9.742e+00	0.0006	1020.0	28.92%	104756.7	26.03%
37	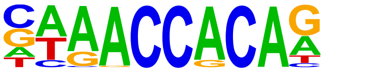	RUNX(Runt)/HPC7-Runx1-ChIP-Seq(GSE22178)/Homer	1e-4	-9.443e+00	0.0008	268.0	7.60%	24220.6	6.02%
38	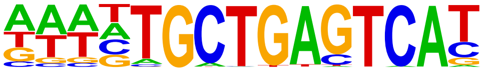	Bach1(bZIP)/K562-Bach1-ChIP-Seq(GSE31477)/Homer	1e-4	-9.385e+00	0.0008	29.0	0.82%	1481.1	0.37%
39	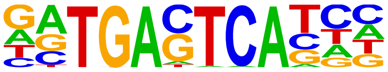	Jun-AP1(bZIP)/K562-cJun-ChIP-Seq(GSE31477)/Homer	1e-4	-9.261e+00	0.0009	98.0	2.78%	7483.7	1.86%
40	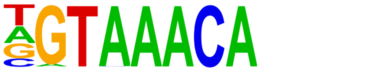	Foxo3(Forkhead)/U2OS-Foxo3-ChIP-Seq(E-MTAB-2701)/Homer	1e-3	-9.193e+00	0.0009	237.0	6.72%	21157.1	5.26%

Supplementary Figure 7: Homer known motif enrichment results for 200-bp regions surrounding CpG sites associated with *DNMT3A* CHIP. *P*-values reflect one-sided binomial tests for enrichment of each motif.

Homer Known Motif Enrichment Results for 200-bp regions surrounding *TET2*-associated CpG sites

Total Target Sequences = 1295, Total Background Sequences = 396414 (after removal of overlapping sequences)

Rank	Motif	Name	P-value	log P-pvalue	q-value (Benjamini)	# Target Sequences with Motif	% of Targets Sequences with Motif	# Background Sequences with Motif	% of Background Sequences with Motif
1	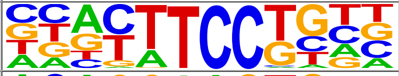	Etv2(ETS)/ES-ER71-ChIP-Seq(GSE59402)/Homer(0.967)	1e-84	-1.947e+02	0.0000	370.0	28.57%	37262.9	9.40%
2	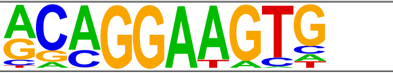	ETS1(ETS)/Jurkat-ETS1-ChIP-Seq(GSE17954)/Homer	1e-82	-1.906e+02	0.0000	400.0	30.89%	43506.3	10.98%
3	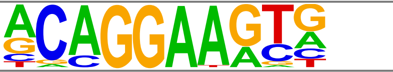	ERG(ETS)/VCaP-ERG-ChIP-Seq(GSE14097)/Homer	1e-80	-1.864e+02	0.0000	510.0	39.38%	67126.0	16.94%
4	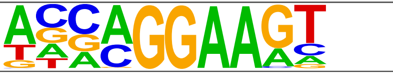	EHF(ETS)/LoVo-EHF-ChIP-Seq(GSE49402)/Homer	1e-61	-1.423e+02	0.0000	392.0	30.27%	50063.4	12.63%
5	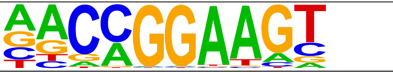	ETV1(ETS)/GIST48-ETV1-ChIP-Seq(GSE22441)/Homer	1e-59	-1.368e+02	0.0000	431.0	33.28%	59605.5	15.04%
6	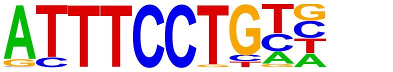	EWS:ERG-fusion(ETS)/CADO_ES1-EWS:ERG-ChIP-Seq(SRA014231)/Homer	1e-58	-1.342e+02	0.0000	258.0	19.92%	25392.1	6.41%
7	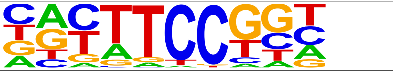	Fli1(ETS)/CD8-FLI-ChIP-Seq(GSE20898)/Homer	1e-56	-1.308e+02	0.0000	391.0	30.19%	52109.6	13.15%
8	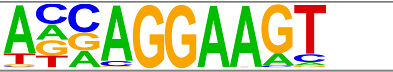	ELF3(ETS)/PDAC-ELF3-ChIP-Seq(GSE64557)/Homer	1e-55	-1.271e+02	0.0000	256.0	19.77%	26023.4	6.57%
9	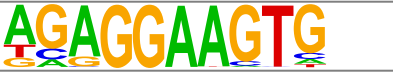	PU.1(ETS)/ThioMac-PU.1-ChIP-Seq(GSE21512)/Homer	1e-54	-1.251e+02	0.0000	204.0	15.75%	17482.0	4.41%
10	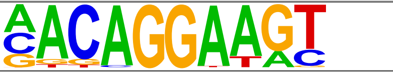	Ets1-distal(ETS)/CD4+-PolII-ChIP-Seq(Barski_et_al.)/Homer	1e-48	-1.118e+02	0.0000	149.0	11.51%	10570.4	2.67%
11	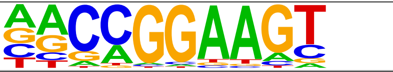	GABPA(ETS)/Jurkat-GABPa-ChIP-Seq(GSE17954)/Homer	1e-43	-9.911e+01	0.0000	298.0	23.01%	38950.4	9.83%
12	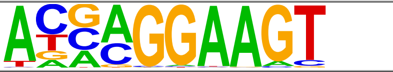	ELF5(ETS)/T47D-ELF5-ChIP-Seq(GSE30407)/Homer	1e-41	-9.487e+01	0.0000	235.0	18.15%	27178.2	6.86%
13	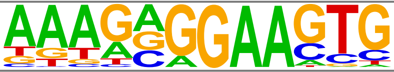	SpiB(ETS)/OCILY3-SPIB-ChIP-Seq(GSE56857)/Homer	1e-31	-7.252e+01	0.0000	105.0	8.11%	8006.2	2.02%
14	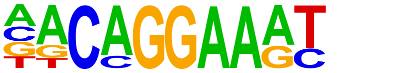	EWS:FLI1-fusion(ETS)/SK_N_MC-EWS:FLI1-ChIP-Seq(SRA014231)/Homer	1e-30	-6.948e+01	0.0000	194.0	14.98%	23873.2	6.02%
15	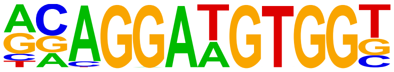	ETS:RUNX(ETS,Runt)/Jurkat-RUNX1-ChIP-Seq(GSE17954)/Homer	1e-23	-5.442e+01	0.0000	62.0	4.79%	3756.7	0.95%
16	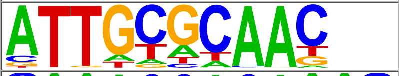	CEBP(bZIP)/ThioMac-CEBPb-ChIP-Seq(GSE21512)/Homer	1e-18	-4.346e+01	0.0000	131.0	10.12%	16710.8	4.22%
17	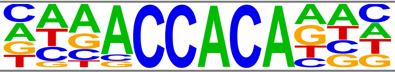	RUNX2(Runt)/PCa-RUNX2-ChIP-Seq(GSE33889)/Homer	1e-17	-4.119e+01	0.0000	185.0	14.29%	28601.7	7.22%
18	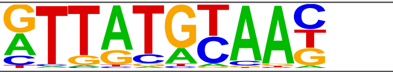	HLF(bZIP)/HSC-HLF.Flag-ChIP-Seq(GSE69817)/Homer	1e-17	-4.078e+01	0.0000	140.0	10.81%	19107.0	4.82%
19	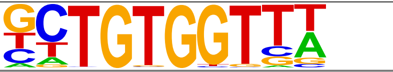	RUNX-AML(Runt)/CD4+-PolII-ChIP-Seq(Barski_et_al.)/Homer	1e-15	-3.553e+01	0.0000	164.0	12.66%	25604.4	6.46%
20	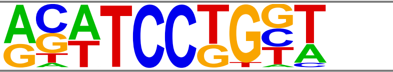	SPDEF(ETS)/VCaP-SPDEF-ChIP-Seq(SRA014231)/Homer	1e-15	-3.516e+01	0.0000	230.0	17.76%	40992.2	10.34%
21	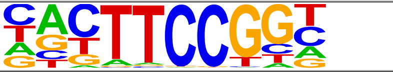	Elk1(ETS)/Hela-Elk1-ChIP-Seq(GSE31477)/Homer	1e-14	-3.351e+01	0.0000	175.0	13.51%	28697.0	7.24%
22	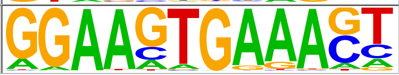	PU.1:IRF8(ETS:IRF)/pDC-Irf8-ChIP-Seq(GSE66899)/Homer	1e-13	-3.062e+01	0.0000	62.0	4.79%	6238.6	1.57%

23	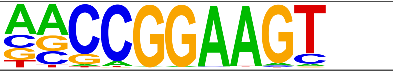	ETS(ETS)/Promoter/Homer	1e-12	-2.990e+01	0.0000	107.0	8.26%	14918.1	3.76%
24	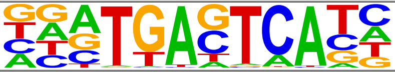	Fra1(bZIP)/BT549-Fra1-ChIP-Seq(GSE46166)/Homer	1e-12	-2.941e+01	0.0000	119.0	9.19%	17556.2	4.43%
25	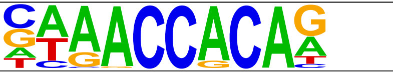	RUNX(Runt)/HPC7-Runx1-ChIP-Seq(GSE22178)/Homer	1e-12	-2.902e+01	0.0000	157.0	12.12%	26073.6	6.58%
26	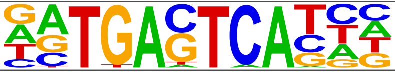	Atf3(bZIP)/GBM-ATF3-ChIP-Seq(GSE33912)/Homer	1e-12	-2.824e+01	0.0000	133.0	10.27%	20929.5	5.28%
27	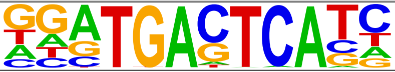	Fra2(bZIP)/Striatum-Fra2-ChIP-Seq(GSE43429)/Homer	1e-12	-2.811e+01	0.0000	109.0	8.42%	15787.2	3.98%
28	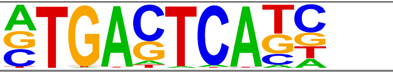	AP-1(bZIP)/ThioMac-PU.1-ChIP-Seq(GSE21512)/Homer	1e-12	-2.766e+01	0.0000	146.0	11.27%	24024.0	6.06%
29	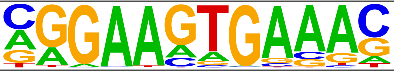	PU.1-IRF(ETS:IRF)/Bcell-PU.1-ChIP-Seq(GSE21512)/Homer	1e-11	-2.698e+01	0.0000	241.0	18.61%	47143.8	11.90%
30	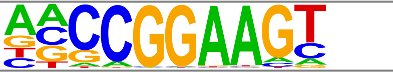	ELF1(ETS)/Jurkat-ELF1-ChIP-Seq(SRA014231)/Homer	1e-11	-2.569e+01	0.0000	148.0	11.43%	25151.1	6.35%
31	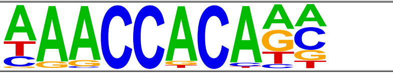	RUNX1(Runt)/Jurkat-RUNX1-ChIP-Seq(GSE29180)/Homer	1e-11	-2.563e+01	0.0000	195.0	15.06%	36342.9	9.17%
32	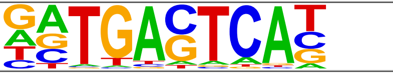	JunB(bZIP)/DendriticCells-Junb-ChIP-Seq(GSE36099)/Homer	1e-10	-2.505e+01	0.0000	113.0	8.73%	17491.3	4.41%
33	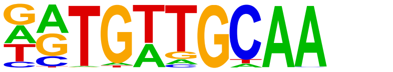	CEBP:AP1(bZIP)/ThioMac-CEBPb-ChIP-Seq(GSE21512)/Homer	1e-10	-2.398e+01	0.0000	128.0	9.88%	21166.0	5.34%
34	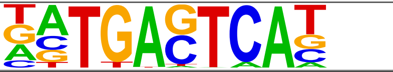	BATF(bZIP)/Th17-BATF-ChIP-Seq(GSE39756)/Homer	1e-10	-2.391e+01	0.0000	123.0	9.50%	20060.0	5.06%
35	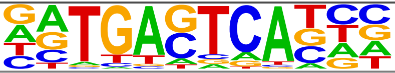	Fosl2(bZIP)/3T3L1-Fosl2-ChIP-Seq(GSE56872)/Homer	1e-10	-2.386e+01	0.0000	82.0	6.33%	11262.5	2.84%
36	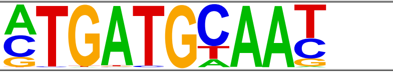	Atf4(bZIP)/MEF-Atf4-ChIP-Seq(GSE35681)/Homer	1e-10	-2.323e+01	0.0000	61.0	4.71%	7288.6	1.84%
37	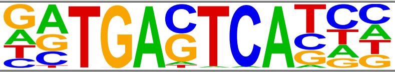	Jun-AP1(bZIP)/K562-cJun-ChIP-Seq(GSE31477)/Homer	1e-9	-2.274e+01	0.0000	65.0	5.02%	8143.6	2.05%
38	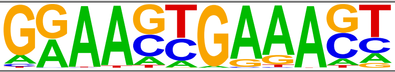	IRF8(IRF)/BMDM-IRF8-ChIP-Seq(GSE77884)/Homer	1e-7	-1.743e+01	0.0000	70.0	5.41%	10416.0	2.63%
39	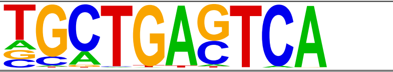	Bach2(bZIP)/OCILy7-Bach2-ChIP-Seq(GSE44420)/Homer	1e-6	-1.536e+01	0.0000	50.0	3.86%	6806.5	1.72%
40	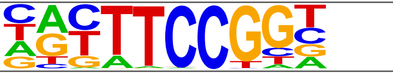	Elk4(ETS)/Hela-Elk4-ChIP-Seq(GSE31477)/Homer	1e-6	-1.514e+01	0.0000	149.0	11.51%	29878.9	7.54%
41	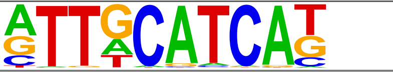	Chop(bZIP)/MEF-Chop-ChIP-Seq(GSE35681)/Homer	1e-6	-1.474e+01	0.0000	43.0	3.32%	5558.0	1.40%
42	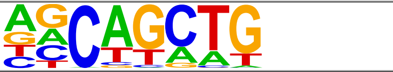	SCL(bHLH)/HPC7-Scl-ChIP-Seq(GSE13511)/Homer	1e-6	-1.394e+01	0.0000	717.0	55.37%	192958.7	48.69%
43	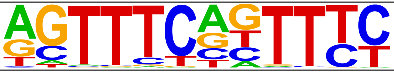	IRF3(IRF)/BMDM-Irf3-ChIP-Seq(GSE67343)/Homer	1e-5	-1.304e+01	0.0000	58.0	4.48%	9108.5	2.30%
44	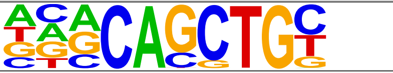	E2A(bHLH)/proBcell-E2A-ChIP-Seq(GSE21978)/Homer	1e-5	-1.297e+01	0.0000	306.0	23.63%	73299.4	18.50%
45	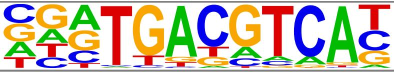	Atf7(bZIP)/3T3L1-Atf7-ChIP-Seq(GSE56872)/Homer	1e-5	-1.218e+01	0.0000	88.0	6.80%	16313.0	4.12%
46	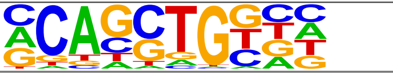	HEB(bHLH)/mES-Heb-ChIP-Seq(GSE53233)/Homer	1e-4	-1.030e+01	0.0003	383.0	29.58%	97765.8	24.67%
47	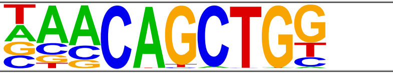	Tcf21(bHLH)/ArterySmoothMuscle-Tcf21-ChIP-Seq(GSE61369)/Homer	1e-4	-1.011e+01	0.0003	186.0	14.36%	42747.8	10.79%
48	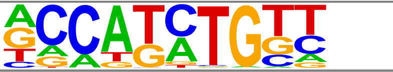	Olig2(bHLH)/Neuron-Olig2-ChIP-Seq(GSE30882)/Homer	1e-4	-9.786e+00	0.0004	294.0	22.70%	72909.0	18.40%
49	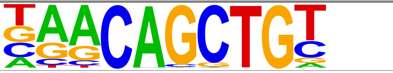	Myf5(bHLH)/GM-Myf5-ChIP-Seq(GSE24852)/Homer	1e-4	-9.247e+00	0.0007	137.0	10.58%	30325.4	7.65%

50	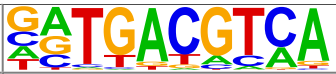	Atf1(bZIP)/K562-ATF1-ChIP-Seq(GSE31477)/Homer	1e-3	-9.104e+00	0.0008	111.0	8.57%	23644.9	5.97%
51	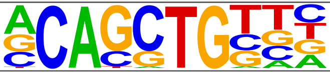	Ptf1a(bHLH)/Panc1-Ptf1a-ChIP-Seq(GSE47459)/Homer	1e-3	-8.953e+00	0.0009	481.0	37.14%	128017.8	32.30%

Supplementary Figure 8: Homer known motif enrichment results for 200-bp regions surrounding *TET2*-associated CpG sites. *P*-values reflect one-sided binomial tests for enrichment of each motif.

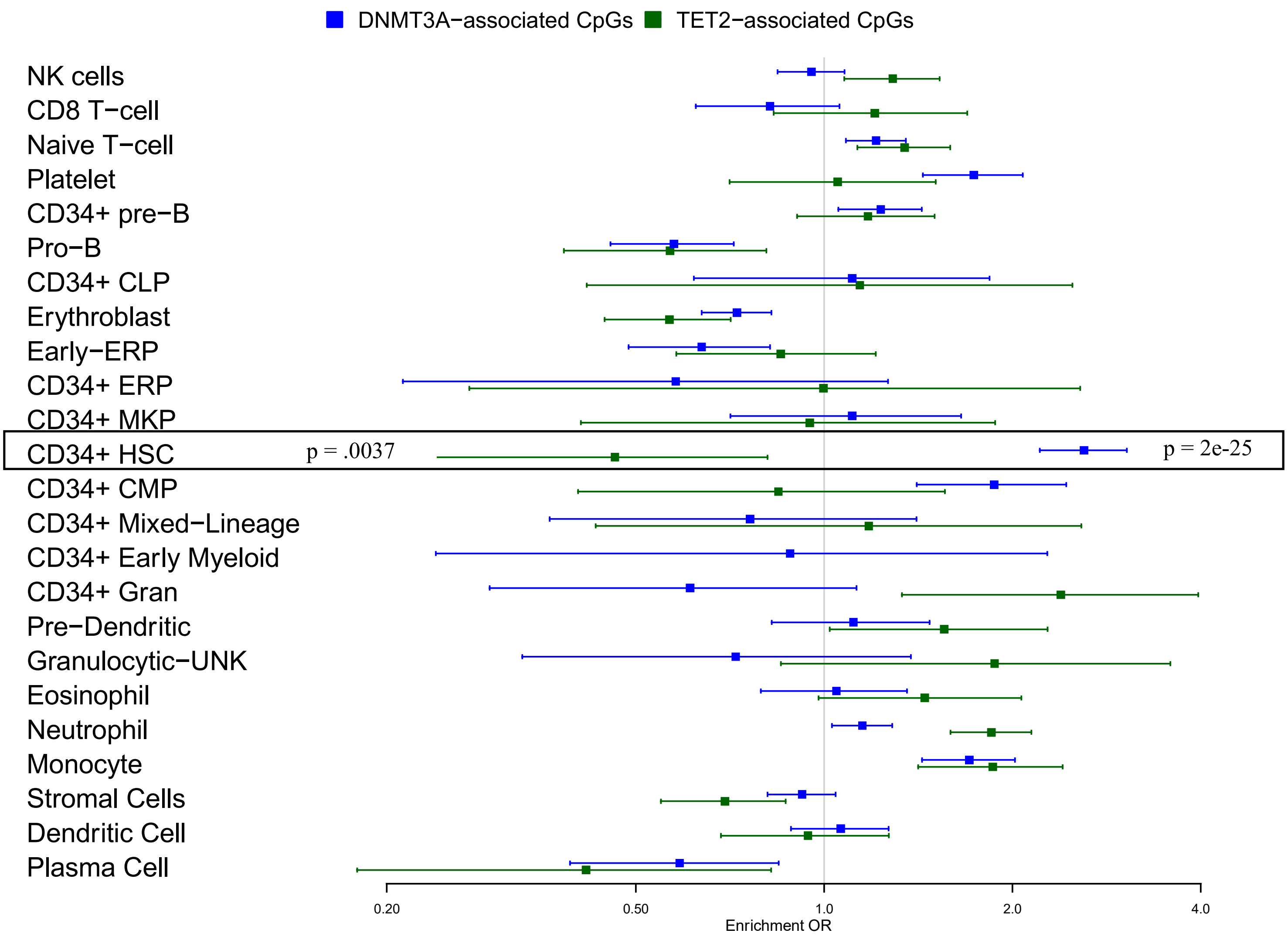

Supplementary Figure 9: Enrichment in human cell-specific gene expression profiles among replicated *DNMT3A*- and *TET2*-associated CpGs. Estimated OR (x-axis, filled squares) shows extent to which genes annotated to *DNMT3A*- or *TET2*-associated CpGs are enriched (or depleted) for sets of marker genes for 24 distinct cell types (y-axis). Marker genes were identified by [10] using Human Cell Atlas scRNA-seq data. Horizontal lines indicate 95% confidence intervals for estimated OR. *P*-values reported in manuscript reflect two-sided Fisher's exact test, and are compared to a Bonferroni-adjusted α-level (.05/48). CLP: common lymphoid progenitor; ERP: erythrocyte progenitor; MKP; megakaryocyte progenitor; CMP: common myeloid progenitor; UNK: unknown.

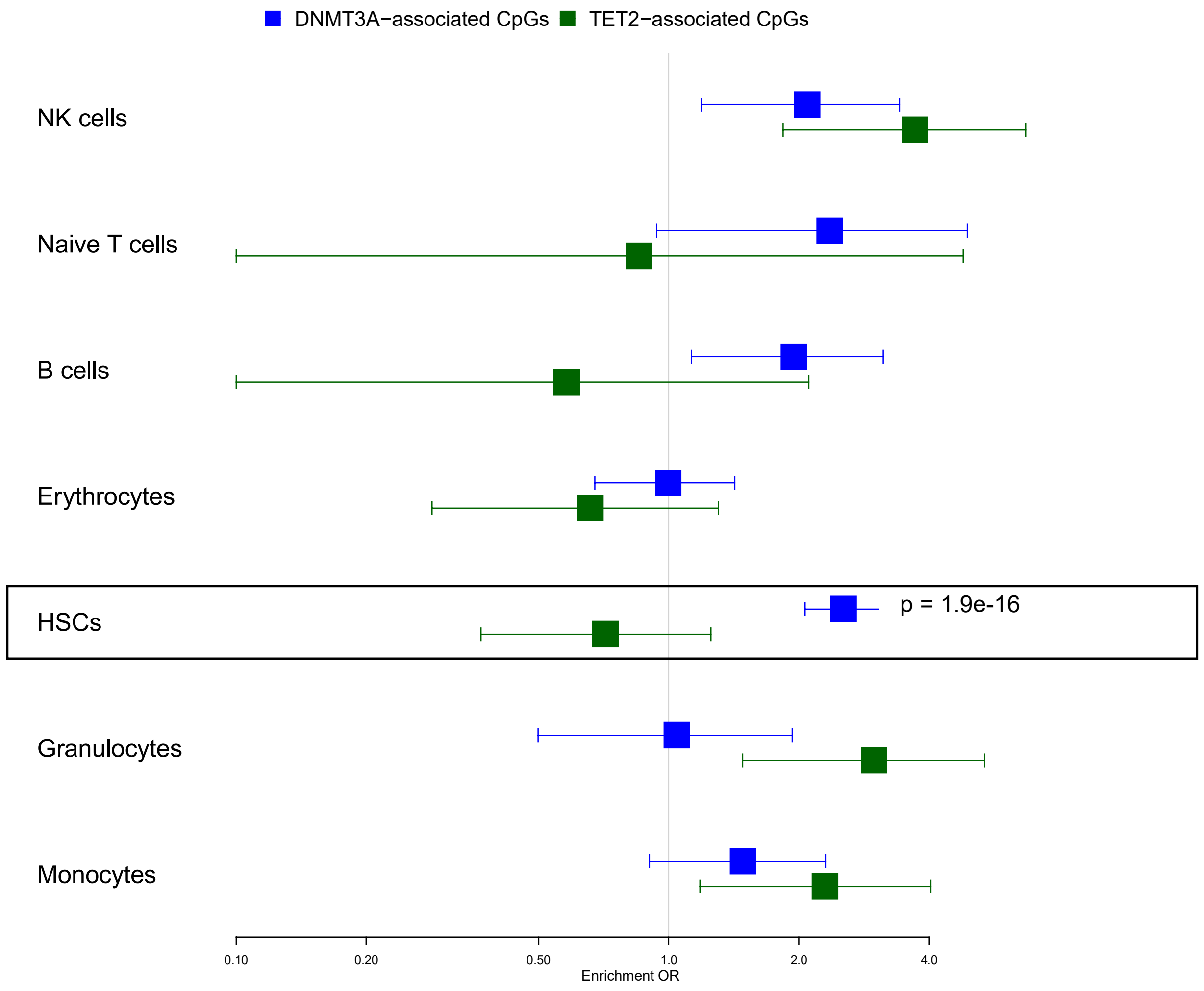

Supplementary Figure 10: Enrichment in murine cell-specific gene expression profiles among replicated *DNMT3A*- and *TET2*-associated CpGs. Estimated OR (x-axis, filled squares) shows extent to which genes annotated to *DNMT3A*- or *TET2*-associated CpGs are enriched (or depleted) for sets of cell-type marker genes identified in mice[11]. *P*-values reported in manuscript reflect two-sided Fisher's exact test, and are compared to a Bonferroni-adjusted α-level (.05/12). Horizontal lines indicate 95% confidence intervals for estimated OR.

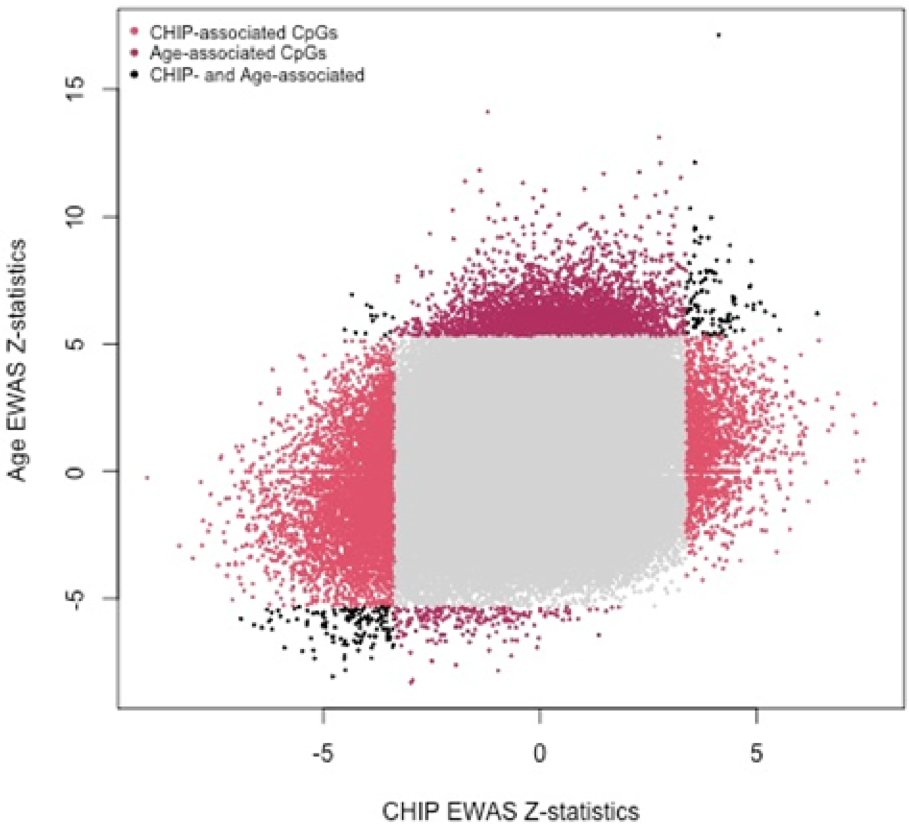

Supplementary Figure 11: Comparison of DNAm profiles associated with CHIP vs. age. CpG-specific Z-statistics from discovery meta-EWAS of CHIP (x-axis) vs. age (y-axis). Point color indicates CpGs associated with CHIP (red), age (maroon) or both (black) according to FDR<0.05.

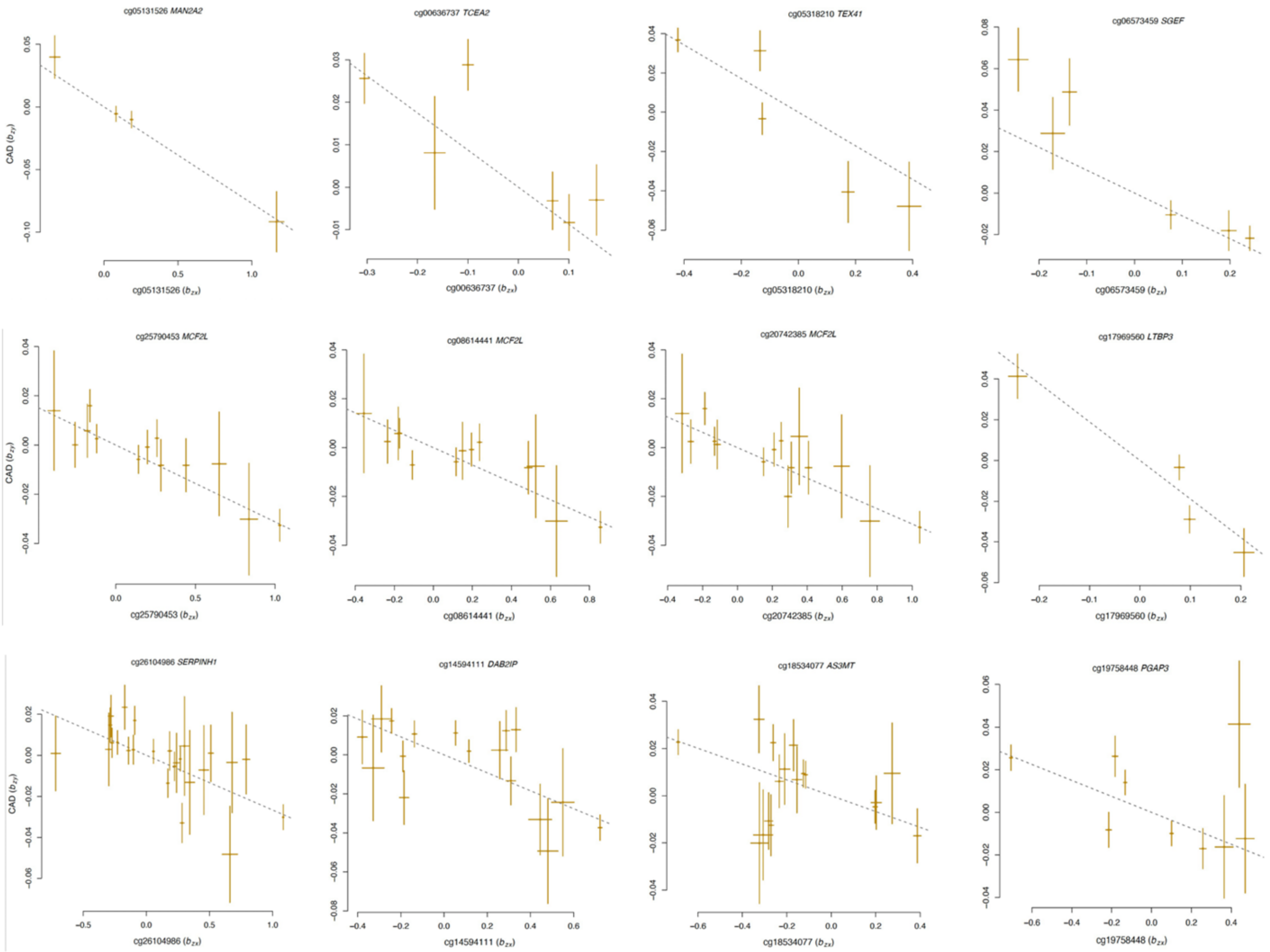

Supplementary Figure 12: Scatterplots depicting SNP effects on exposures (CpGs) vs. SNP effects on outcome (CAD). Effect sizes of partially independent (LD, r^2 <0.05) SNPs for *cis*-mQTL (b_{zx}) on the x-axis and for coronary artery disease GWAS (b_{zy}) on the y-axis. Vertical lines represent standard error bars (±1 SE) for b_{zy}, and the dotted line represents the slope of the best fitted line (b_{xy}). Only Bonferroni significant (P<0.05/2580) GSMR results presented here. CAD=coronary artery disease; mQTL=methylation quantitative trait loci.

Supplementary Tables

Supplementary Table 1: Eleven CpGs common in the Any CHIP, Expanded CHIP (variant allele fraction>10%), *DNMT3A*, and *TET2* CHIP meta EWAS. Inverse variance weighted fixed effect meta-analysis was performed using METAL software[8]. Here, "Direction" column (e.g. "----") represents the effect direction in corresponding EWAS in CHS AA, CHS EA, ARIC AA, and ARIC EA, respectively. AA: African ancestry; EA: European ancestry. Significance was defined as FDR<.05, based on two-sided *P*-values.

**CpG**	**CHR**	**POS (hg19)**	**Gene**	**Any CHIP**				**Expanded CHIP**				***DNMT3A* CHIP**				***TET2* CHIP**			
				**Beta**	**SE**	***P***	**Direction**	**Beta**	**SE**	***P***	**Direction**	**Beta**	**SE**	***P***	**Direction**	**Beta**	**SE**	***P***	**Direction**
cg04134748	1	3052175	*PRDM16*	-0.043	0.0052	1.4E-16	----	-0.061	0.0062	3.1E-22	----	-0.050	0.0063	2.5E-15	----	-0.087	0.0140	6.5E-10	----
cg17859426	3	113667809	*ZDHHC23*	-0.028	0.0039	7.9E-13	----	-0.034	0.0046	2.2E-13	----	-0.026	0.0047	3.6E-08	----	-0.046	0.0104	1.1E-05	----
cg08234504	5	139013317		-0.017	0.0019	3.3E-18	----	-0.022	0.0023	4.3E-22	----	-0.016	0.0024	1.2E-11	----	-0.023	0.0046	5.5E-07	---+
cg24803517	5	94621517	*MCTP1*	-0.040	0.0050	2.3E-15	----	-0.050	0.0059	1.1E-17	----	-0.034	0.0062	5.7E-08	----	-0.065	0.0118	3.1E-08	---+
cg05302489	6	31760426	*VARS*	-0.036	0.0044	6.3E-16	----	-0.049	0.0051	2.1E-21	----	-0.030	0.0056	9.6E-08	----	-0.051	0.0104	9.4E-07	---+
cg08268017	8	94712118	*FAM92A1*	-0.030	0.0040	7.5E-14	----	-0.038	0.0047	1.8E-15	----	-0.029	0.0048	1.5E-09	----	-0.049	0.0097	3.9E-07	---+
cg13303654	10	31610754	*ZEB1*	-0.035	0.0048	6.2E-13	----	-0.043	0.0057	6.9E-14	----	-0.032	0.0060	6.4E-08	----	-0.055	0.0122	6.3E-06	---+
cg22266877	11	64058550	*KCNK4*	-0.020	0.0033	5.3E-10	----	-0.027	0.0039	2.4E-12	----	-0.016	0.0039	5.7E-05	----	-0.039	0.0076	3.4E-07	---+
cg20558112	13	45968589	*SLC25A30*	-0.028	0.0031	5.6E-20	----	-0.035	0.0037	2.1E-21	----	-0.026	0.0038	2.5E-12	----	-0.043	0.0074	6.4E-09	---+
cg18454133	14	23835870	*EFS*	-0.013	0.0016	1.5E-14	----	-0.019	0.0020	1.5E-20	----	-0.012	0.0019	2.8E-11	----	-0.027	0.0046	2.7E-09	---+
cg19325791	17	46560683		-0.027	0.0034	8.4E-15	----	-0.035	0.0040	1.2E-18	----	-0.021	0.0043	6.0E-07	----	-0.045	0.0082	3.0E-08	----

Supplementary Table 2: Enrichment of FDR Significant CpGs in the UCSC annotated CpG Island categories. *P*-values reflect two-sided Fisher's exact test was performed, and are compared to a Bonferroni-adjusted α-level (.05/12).

**CHIP category**	**Relation to CpG Island**	**N CpGs**	**N DM CpGs**	**OR**	**95% CI**	**P**
Any CHIP	Open Sea	172094	1556	0.82	0.77-0.87	8.0E-11
Any CHIP	Island	149620	689	0.36	0.33-0.39	9.3E-175
Any CHIP	Shore	110829	2254	2.83	2.68-3	2.5E-267
Any CHIP	Shelf	46116	413	0.86	0.77-0.95	3.0E-03
DNMT3A	Open Sea	172094	1691	1.42	1.33-1.52	1.6E-26
DNMT3A	Island	149616	299	0.19	0.16-0.21	1.2E-270
DNMT3A	Shore	110834	1395	1.93	1.8-2.06	4.0E-78
DNMT3A	Shelf	46117	418	1.16	1.04-1.28	5.3E-03
TET2	Open Sea	172096	850	2.41	2.17-2.67	3.7E-63
TET2	Island	149616	100	0.16	0.13-0.19	3.0E-118
TET2	Shore	110829	284	0.79	0.69-0.9	2.4E-04
TET2	Shelf	46120	245	1.87	1.62-2.14	1.2E-16

Supplementary Table 3: Enrichment of FDR-Significant CpGs in Differentially Methylated Regions (DMRs) previously associated with colorectal cancer, tissue type, or reprogramming. *P*-values reflect two-sided Fisher's exact test was performed, and are compared to a Bonferroni-adjusted α-level (.05/9). C-DMR= Cancer-specific DMR; R-DMR = Reprogramming-specific DMR.

**CHIP category**	**DMR**	**N CpGs**	**N DM CpGs**	**OR**	**95% CI**	***P***
Any CHIP	C-DMR	6573	120	1.81	1.5-2.18	5.1E-09
Any CHIP	T-DMR	18353	70	0.36	0.28-0.46	1.2E-23
Any CHIP	R-DMR	12086	370	3.21	2.87-3.58	7.1E-75
DNMT3A	C-DMR	6573	85	1.65	1.31-2.05	2.4E-05
DNMT3A	T-DMR	18353	38	0.25	0.18-0.35	6.2E-27
DNMT3A	R-DMR	12086	276	3.07	2.7-3.47	4.9E-53
TET2	C-DMR	6573	17	0.83	0.48-1.34	5.7E-01
TET2	T-DMR	18353	17	0.29	0.17-0.47	6.6E-10
TET2	R-DMR	12086	28	0.74	0.49-1.08	1.3E-01

Supplementary Table 4: Genes with ten or more FDR Significant CpGs. One-sided Fisher's exact test was performed using data from Supplementary Data 1-3, 9. Genes with OR>1 and *P*<0.05 were reported in the table.

**CHIP Category**	**Gene**	**No. CpGs**	**Total CpGs in Gene**	**Odds Ratio**	***P***
Any CHIP	*PRDM16* (NM_022114)	28	640	4.4	3.1E-10
Any CHIP	*VARS* (NM_006295)	14	104	15.0	4.7E-12
Any CHIP	*PPT2* (NM_138717); *PRRT1*	14	107	14.6	7.0E-12
Any CHIP	*AGAP2* (NM_014770)	13	38	50.2	5.9E-17
Any CHIP	*MCF2L* (NM_001112732)	13	301	4.4	1.8E-05
Any CHIP	*BCL9L* (NM_182557)	10	23	74.4	1.3E-14
Any CHIP	*PEX10* (NM_002617)	10	47	26.1	4.7E-11
Any CHIP	*PPP1R18* (NM_133471)	10	103	10.4	1.3E-07
Any CHIP	*PLEC1* (NM_000445)	10	154	6.7	5.2E-06
Any CHIP	*GABBR1* (NM_001470)	10	185	5.5	2.6E-05
Any CHIP	*C7orf50* (NM_032350)	10	323	3.1	2.1E-03
Expanded CHIP	*PRDM16* (NM_022114)	21	640	3.8	4.7E-07
Expanded CHIP	*AGAP2* (NM_014770)	14	38	65.7	1.4E-19
Expanded CHIP	*PPT2* (NM_138717); *PRRT1*	13	107	15.6	1.6E-11
Expanded CHIP	*VARS* (NM_006295)	12	104	14.7	1.8E-10
Expanded CHIP	*MCF2L* (NM_001112732)	10	301	3.9	4.1E-04
Expanded CHIP	*C7orf50* (NM_032350)	10	323	3.6	7.0E-04
DNMT3A	*PRDM16* (NM_022114)	36	640	7.5	2.2E-19
DNMT3A	*PPT2* (NM_138717); *PRRT1*	16	107	22.1	5.4E-16
DNMT3A	*HOXB3* (NM_002146)	12	55	35.0	2.0E-14
DNMT3A	*VARS* (NM_006295)	12	104	16.3	5.5E-11
DNMT3A	*SEPT9* (NM_001113491)	11	166	8.9	1.2E-07
TET2	*RPTOR* (NM_020761)	18	435	14.1	6.1E-15
TET2	*HDAC4* (NM_006037)	15	419	12.1	8.4E-12
TET2	*TRIM39* (NM_021253)	10	125	28.2	9.9E-12

Supplementary References

1 Agha, G. *et al.* Blood Leukocyte DNA Methylation Predicts Risk of Future Myocardial Infarction and Coronary Heart Disease. *Circulation* **140**, 645-657, doi:10.1161/CIRCULATIONAHA.118.039357 (2019).

2 Aryee, M. J. *et al.* Minfi: a flexible and comprehensive Bioconductor package for the analysis of Infinium DNA methylation microarrays. *Bioinformatics* **30**, 1363-1369, doi:10.1093/bioinformatics/btu049 (2014).

3 Maksimovic, J., Gordon, L. & Oshlack, A. SWAN: Subset-quantile within array normalization for illumina infinium HumanMethylation450 BeadChips. *Genome Biol* **13**, R44, doi:10.1186/gb-2012-13-6-r44 (2012).

4 Houseman, E. A. *et al.* DNA methylation arrays as surrogate measures of cell mixture distribution. *BMC Bioinformatics* **13**, 86, doi:1471-2105-13-86 [pii]
10.1186/1471-2105-13-86 (2012).

5 Demerath, E. W. *et al.* Epigenome-wide association study (EWAS) of BMI, BMI change and waist circumference in African American adults identifies multiple replicated loci. *Hum Mol Genet* **24**, 4464-4479, doi:10.1093/hmg/ddv161 (2015).

6 Triche, T. J., Jr., Weisenberger, D. J., Van Den Berg, D., Laird, P. W. & Siegmund, K. D. Low-level processing of Illumina Infinium DNA Methylation BeadArrays. *Nucleic Acids Res* **41**, e90, doi:gkt090 [pii]
10.1093/nar/gkt090 (2013).

7 Teschendorff, A. E. *et al.* A beta-mixture quantile normalization method for correcting probe design bias in Illumina Infinium 450 k DNA methylation data. *Bioinformatics* **29**, 189-196, doi:10.1093/bioinformatics/bts680 (2013).

8 Willer, C. J., Li, Y. & Abecasis, G. R. METAL: fast and efficient meta-analysis of genomewide association scans. *Bioinformatics* **26**, 2190-2191, doi:10.1093/bioinformatics/btq340 (2010).

9 Lex, A., Gehlenborg, N., Strobelt, H., Vuillemot, R. & Pfister, H. UpSet: Visualization of Intersecting Sets. *IEEE Trans Vis Comput Graph* **20**, 1983-1992, doi:10.1109/TVCG.2014.2346248 (2014).

10 Hay, S. B., Ferchen, K., Chetal, K., Grimes, H. L. & Salomonis, N. The Human Cell Atlas bone marrow single-cell interactive web portal. *Exp Hematol* **68**, 51-61, doi:10.1016/j.exphem.2018.09.004 (2018).

11 Chambers, S. M. *et al.* Hematopoietic fingerprints: an expression database of stem cells and their progeny. *Cell Stem Cell* **1**, 578-591, doi:10.1016/j.stem.2007.10.003 (2007).
