# Supplementary material for: Clonal hematopoiesis of indeterminate potential, DNA methylation, and risk for coronary artery disease
Source: Nat Commun. 2022 Sep 12;13:5350. doi: 10.1038/s41467-022-33093-3 (PMC9468335; doi:10.1038/s41467-022-33093-3)
Supplement: Supplementary file 3 — Description of Additional Supplementary Files [file 41467_2022_33093_MOESM3_ESM.pdf]

Description of Additional Supplementary Files

File Name: Supplementary Data 1
Description: **EWAS summary statistics for any CHIP associated replicated CpGs.** Summary statistics include meta-analysis of CHS AA and EA EWAS, ARIC AA and EA EWAS, and combined meta EWAS of CHS AA, CHS EA, ARIC AA and ARIC EA (Effect direction follows the same order). AA: African ancestry; EA: European ancestry; HetISq: heterogeneity I^2; HetP: heterogeneity *P*-value. Significance was defined as FDR<.05, based on two-sided *P*-values from inverse variance weighted fixed effect meta-analysis.

File Name: Supplementary Data 2
Description: **EWAS summary statistics for *DNMT3A* CHIP associated replicated CpGs.** Summary statistics include meta-analysis of CHS AA and EA EWAS, ARIC AA and EA EWAS, and combined meta EWAS of CHS AA, CHS EA, ARIC AA and ARIC EA (Effect direction follows the same order). AA: African ancestry; EA: European ancestry; HetISq: heterogeneity I^2; HetP: heterogeneity *P*-value. Significance was defined as FDR<.05, based on two-sided *P*-values from inverse variance weighted fixed effect meta-analysis.

File Name: Supplementary Data 3
Description: **EWAS summary statistics for *TET2* CHIP associated replicated CpGs.** Summary statistics include meta-analysis of CHS AA and EA EWAS, ARIC AA and EA EWAS, and combined meta EWAS of CHS AA, CHS EA, ARIC AA and ARIC EA (Effect direction follows the same order). AA: African ancestry; EA: European ancestry; HetISq: heterogeneity I^2; HetP: heterogeneity *P*-value. Significance was defined as FDR<.05, based on two-sided *P*-values from inverse variance weighted fixed effect meta-analysis.

File Name: Supplementary Data 4
Description: **EWAS summary statistics for the *TET2* CHIP associated CpGs replicated in Tulstrup et. al. 2021.** Summary statistics include meta-analysis of CHS AA and EA EWAS (Effect direction follows the same order). CpG annotations from Illumina 450k manifest HumanMethylation450_15017482_v1-2.csv. AA: African ancestry; EA: European ancestry; HetISq: heterogeneity I^2; HetP: heterogeneity *P*-value. Significance was defined as FDR<.05, based on two-sided *P*-values from inverse variance weighted fixed effect meta-analysis.

File Name: Supplementary Data 5
Description: **Gene Ontology (GO) enrichment for replicated *DNMT3A* CHIP associated CpGs.** The missmethyl R package was used for GO enrichment analysis. This analysis tested for enrichment of 22,710 GO terms: 15,988 biological process (BP), 1,979 cellular component (CC), and 4,743 molecular function (MF) related terms using a Wallenius' non-central hypergeometric test (one-sided). *N* Genes: number of genes in the GO term; *N* DM: number of genes that are differentially methylated; *P*: p-value for over-representation of the GO term; FDR: false discovery rate.

File Name: Supplementary Data 6
Description: **GO enrichment for replicated *TET2* CHIP associated CpGs. The missmethyl R package was used for GO enrichment analysis.** The missmethyl R package was used for GO enrichment analysis Wallenius' non-central hypergeometric test (one-sided). This analysis tested

for enrichment of 22,710 GO terms: 15,988 biological process (BP), 1,979 cellular component (CC), and 4,743 molecular function (MF) related terms. *N* Genes: number of genes in the GO term; *N* DM: number of genes that are differentially methylated; *P*: p-value for over-representation of the GO term; FDR: false discovery rate.

File Name: Supplementary Data 7
Description: **Summary statistics of Mendelian randomization between *cis*-mQTL and CAD.** GCTA software GSMR package was used for the MR analysis. mQTL=methylation quantitative trait loci; CAD=coronary artery disease. GSMR analysis was based on published summary statistics (effect estimates) for cis-mQTL[1] (*N*=32,851) and CAD GWAS[2] (*N*=547,261). Significance was defined as FDR<0.05 based on *P*-values from 2-sided χ^2 tests.

File Name: Supplementary Data 8
Description: **Partially independent (LD r^2<0.05) *cis*-mQTL and *cis*-eQTL (Bonferroni adjusted P <0.05) summary statistics from Min, et al. [1] (http://mqtldb.godmc.org.uk/) and Vosa, et al. [3] (https://www.eqtlgen.org/), respectively.** These cis-mQTL (P<5e-8) were used as instrumental variables in Mendelian randomization in GSMR analysis. mQTL=methylation quantitative trait loci; eQTL=expression quantitative trait loci.

File Name: Supplementary Data 9
Description: **EWAS summary statistics for expanded CHIP (variant allele fraction >10%) associated replicated CpGs.** Summary statistics include meta-analysis of CHS AA and EA EWAS, ARIC AA and EA EWAS, and combined meta EWAS of CHS AA, CHS EA, ARIC AA and ARIC EA (Effect direction follows the same order). AA: African ancestry; EA: European ancestry; HetISq: heterogeneity I^2; HetP: heterogeneity *P*-value. Significance was defined as FDR<.05, based on two-sided *P*-values from inverse variance weighted fixed effect meta-analysis.
