# Supplementary material for: Clonal hematopoiesis of indeterminate potential, DNA methylation, and risk for coronary artery disease
Source: Nat Commun. 2022 Sep 12;13:5350. doi: 10.1038/s41467-022-33093-3 (PMC9468335; doi:10.1038/s41467-022-33093-3)
Supplement: Supplementary file 5 — Reporting Summary [file 41467_2022_33093_MOESM5_ESM.pdf]

nature portfolio

Corresponding author(s): Karen Conneely, Pradeep Natarajan

Last updated by author(s): Aug 22, 2022

Reporting Summary

Nature Portfolio wishes to improve the reproducibility of the work that we publish. This form provides structure for consistency and transparency in reporting. For further information on Nature Portfolio policies, see our Editorial Policies and the Editorial Policy Checklist.

Statistics

For all statistical analyses, confirm that the following items are present in the figure legend, table legend, main text, or Methods section.

n/a	Confirmed	
☐	☒	The exact sample size (*n*) for each experimental group/condition, given as a discrete number and unit of measurement
☐	☒	A statement on whether measurements were taken from distinct samples or whether the same sample was measured repeatedly
☐	☒	The statistical test(s) used AND whether they are one- or two-sided<br>*Only common tests should be described solely by name; describe more complex techniques in the Methods section.*
☐	☒	A description of all covariates tested
☐	☒	A description of any assumptions or corrections, such as tests of normality and adjustment for multiple comparisons
☐	☒	A full description of the statistical parameters including central tendency (e.g. means) or other basic estimates (e.g. regression coefficient) AND variation (e.g. standard deviation) or associated estimates of uncertainty (e.g. confidence intervals)
☐	☒	For null hypothesis testing, the test statistic (e.g. *F*, *t*, *r*) with confidence intervals, effect sizes, degrees of freedom and *P* value noted<br>*Give P values as exact values whenever suitable.*
☒	☐	For Bayesian analysis, information on the choice of priors and Markov chain Monte Carlo settings
☒	☐	For hierarchical and complex designs, identification of the appropriate level for tests and full reporting of outcomes
☐	☒	Estimates of effect sizes (e.g. Cohen's *d*, Pearson's *r*), indicating how they were calculated

Our web collection on statistics for biologists contains articles on many of the points above.

Software and code

Policy information about availability of computer code

Data collection	DNA methylation data were processed using R software v4.0, with specific R packages and functions described in Supplemental Methods. CHIP data were processed using the following software, with numbered references corresponding to the main text bibliography: CHIP was detected previously in CHS from WGS blood DNA in the NHLBI Trans-Omics for Precision Medicine consortium[73]. The same procedure was applied for WES data in ARIC. Mutect2 software[75] was used for somatic mutation calling from WGS data in CHS and WES data in ARIC. CHIP was called from the Annovar annotated VCF files using a custom R script and predefined list of CHIP genes, variants, and rules. The detailed CHIP calling pipeline was previously reported in Bick, et al. [73] (https://app.terra.bio/#workspaces/terra-outreach/CHIP-Detection-Mutect2).
Data analysis	Except for the following four exceptions, all data analysis was performed using R software v4.1.1, with specific packages and functions referenced in the text. Exception 1) METAL software[78] (2020-05-05 version) was used to perform inverse variance weighted fixed effect meta-analysis and Cochran's Q-test for heterogeneity[79]. Exception 2) We used the HOMER software suite[26] v4.11 to test the 200-bp regions surrounding replicated CpGs for enrichment for previously reported transcription factor binding motifs. Exception 3) We used the eFORGE tool[32] v2.0 to test the top 1000 CpG sites in each set for enrichment in regions identified as DNase I hypersensitive (DHS) hotspots generated by the ENCODE project[33]. All of these are described in the text, and an archive of our code is available on Github: https://github.com/MMesbahU/CHIP-EWAS [86; doi:10.5281/zenodo.7007412]. Exception 4) We used GCTA v1.93.2[84,85] for the Mendelian randomization analysis.

For manuscripts utilizing custom algorithms or software that are central to the research but not yet described in published literature, software must be made available to editors and reviewers. We strongly encourage code deposition in a community repository (e.g. GitHub). See the Nature Portfolio guidelines for submitting code & software for further information.

Data

Policy information about availability of data

All manuscripts must include a data availability statement. This statement should provide the following information, where applicable:

- Accession codes, unique identifiers, or web links for publicly available datasets
- A description of any restrictions on data availability
- For clinical datasets or third party data, please ensure that the statement adheres to our policy

To protect the privacy of research participants and the confidentiality of their data while ensuring that these data are available for appropriate use by researchers, all raw data used in this study are available via controlled access. Individual whole genome sequencing data for CHS whole genomes generated via TOPMed and the CHIP somatic variant call sets are available through controlled access via dbGaP (https://www.ncbi.nlm.nih.gov/gap/) accession code phs001368. Individual whole exome sequencing data from ARIC are available via dbGaP accession code phs000668. DNA methylation data, as well as phenotypic data, are available via controlled access via ancillary study proposals. Timelines for the approval process range from 4-9 weeks for CHS and 3-6 weeks for ARIC ancillary studies, with specific criteria and proposal forms for the respective studies available at https://chs-nhlbi.org/node/6222 and https://sites.cscc.unc.edu/aric/ancillary-studies-pfg. Summary statistics for replicated associations are available in supplementary tables, and full discovery EWAS summary statistics are available from the Downloads page of the Cardiovascular Disease Knowledge Portal (CVDKP; https://cvd.hugeamp.org/downloads.html). For enrichment analyses, WGBS data from BLUEPRINT were downloaded from from GEO series GSE87196, murine marker genesets were obtained from Ensembl Release 10582, and tumor DNAm data from TCGA were downloaded from https://gdc.cancer.gov/about-data/publications/laml_2012.

Field-specific reporting

Please select the one below that is the best fit for your research. If you are not sure, read the appropriate sections before making your selection.

☒ Life sciences ☐ Behavioural & social sciences ☐ Ecological, evolutionary & environmental sciences

For a reference copy of the document with all sections, see nature.com/documents/nr-reporting-summary-flat.pdf

Life sciences study design

All studies must disclose on these points even when the disclosure is negative.

Sample size	The sample size was determined by the inclusion of all CHS participants for whom both CHIP data and DNA methylation data were available.
Data exclusions	CHS samples were excluded if (i) median intensities across the methylated and unmethylated channels were <10.5 (log2), (ii) >0.5% of probes failed detection, (iii) QC probes fell >3 SD from the mean, (iv) sample swaps appeared likely due to sex mismatches or genotype inconsistency with prior genotyping. ARIC individuals were excluded from the analyses if the pass rate for the DNA sample for the participant was <95% (number of probes with a detection p-value <0.01/number of probes on the array), if sex mismatch appeared likely based on principal component analysis, or if genotypes for 24 single nucleotide polymorphisms present on the array were inconsistent with prior genotyping.
Replication	The CHS sample was used for discovery, and an independent replication study was carried out in 2655 participants from the Atherosclerosis Risk in Communities (ARIC) Study. For the three sets of CpG sites significant in discovery analysis, 66%, 84%, and 13% showed successful replication in ARIC.
Randomization	Because this was an observational population-based study, no experimental manipulations were applied. Random design was used in the assignment of individual samples to DNA methylation chips.
Blinding	Because this was an observational population-based study, no blinding was required. However, the use of standard statistical analyses and pipelines applied via computer code effectively blinds the researchers to the sample characteristics during analysis.

Reporting for specific materials, systems and methods

We require information from authors about some types of materials, experimental systems and methods used in many studies. Here, indicate whether each material, system or method listed is relevant to your study. If you are not sure if a list item applies to your research, read the appropriate section before selecting a response.

Materials & experimental systems

n/a	Involved in the study	
☒	☐	Antibodies
☒	☐	Eukaryotic cell lines
☒	☐	Palaeontology and archaeology
☒	☐	Animals and other organisms
☐	☒	Human research participants
☒	☐	Clinical data
☒	☐	Dual use research of concern

Methods

n/a	Involved in the study	
☒	☐	ChIP-seq
☒	☐	Flow cytometry
☒	☐	MRI-based neuroimaging

Human research participants

Policy information about studies involving human research participants

Population characteristics	In CHS, 61% of participants were female, 48% were African American, and the mean (standard deviation) age was 73.6 (5.2) years at the time of blood draw for whole genome sequencing (WGS). In ARIC, 61% of participants were female, 71% were African-American, and the mean (standard deviation) age was 57.4 (5.9) years at the time of blood draw for whole exome sequencing (WES).
Recruitment	We analyzed data on individuals previously recruited by the Cardiovascular Health Study, with recruitment described in Fried et al. [74], and the Atherosclerosis Risk in Communities (ARIC) Study, described in doi:10.1093/hmg/ddv161.
Ethics oversight	Informed consent for genetic studies was obtained from all participants, and study protocols were approved by the respective institutional review board (IRB) for each cohort (University of Washington Institutional Review Board (CHS); University of Mississippi Medical Center Institutional Review Board (ARIC: Jackson Field Center); Wake Forest University Health Sciences Institutional Review Board (ARIC: Forsyth County Field Center); University of Minnesota Institutional Review Board (ARIC: Minnesota Field Center); and Johns Hopkins University School of Public Health Institutional Review Board (ARIC: Washington County Field Center). Each study received institutional certification before depositing sequencing data into dbGaP, ensuring approval by all relevant institutional ethics committees and compliance with relevant ethical regulations.

Note that full information on the approval of the study protocol must also be provided in the manuscript.
